# STROBE-compliant article: Blood Transfusions within the First 24 Hours of Hospitalization Did Not Impact Mortality Among Patients with Severe Sepsis

**DOI:** 10.1097/MD.0000000000002601

**Published:** 2016-01-29

**Authors:** Chih-Yi Hsu, Su-Hsun Liu, Chung-Hsien Chao, Yi-Lin Chan, Tsung-Cheng Tsai, Li-Min Chen, Chin-Chieh Wu, Kuan-Fu Chen

**Affiliations:** From the Department of Emergency Medicine, Chang Gung Memorial Hospital, Kaohsiung (C-YH, T-CT); Department of Family Medicine, Chang Gung Memorial Hospital, Linkou (S-HL); Department of Emergency Medicine, Chang Gung Memorial Hospital, Linkou (C-HC, Y-LC); School of Medicine, Chang Gung University, Taoyuan (S-HL); Department of Emergency Medicine, Chang Gung Memorial Hospital, Keelung (L-MC, C-CW, K-FC); Community Medicine Research Center, Chang Gung Memorial Hospital, Keelung (K-FC); and Clinical Informatics and Medical Statistics Research Center, Chang Gung University, Taoyuan, Taiwan (K-FC).

## Abstract

Transfusion of packed red blood cells is common during resuscitation of critically ill patients. However, the association between in-hospital mortality and blood transfusion among patients with severe sepsis during the first 24 hours of hospitalization has not yet been determined.

A cohort study was conducted of adult nontrauma patients who visited the emergency department of a tertiary hospital and were diagnosed with severe sepsis. Propensity score (PS) matching was conducted, based on patient demographics, underlying illnesses, laboratory results, and vital signs presented at the emergency department, and multivariate logistic regression was performed to adjust for potential residual confounding between the 2 transfused and nontransfused groups to assess the risk of in-hospital mortality.

Of 3448 patients included in this study, 265 underwent blood transfusion during the first 24 hours of hospitalization. Despite comparable severity of sepsis, patients who received transfusions tended to have lower mean arterial pressures (86 vs 98 mmHg) and hemoglobin levels (7.6 vs 11.2 g/dL), and were more likely to have chronic kidney disease (12% vs 6%) and hematologic organ dysfunction (57% vs 35%, all *P* < 0.001). Transfused patients tended to have higher mortality rates (26% vs 9%, respectively, *P* *<* 0.001). After PS matching, 177 pairs of transfused and nontransfused patients were analyzed. After adjusting for residual confounding factors by multivariate logistic regression in the matched patient pairs, no significant differences in in-hospital mortality were observed (odds ratio [OR] = 1.52, 95% confidence interval: 0.92–2.51).

In this PS-matched cohort study of adult nontrauma patients with severe sepsis, the in-hospital mortality rate was not significantly different in patients who received blood transfusions during the first 24 hours of hospitalization.

## Abstract 2

Supplemental Digital Content is available in the text

## INTRODUCTION

Sepsis, one of the most common diseases presented in the emergency department (ED) that requires significant medical resources and attention, remains the primary cause of death from infection.^1^ In Taiwan, the incidence rates of severe sepsis increased 1.6-fold, from 135 per 100,000 in 1997 to 217 per 100,000 in 2006, with an annual percent change of 3.9%.^2^ Immediate recognition and early high-quality resuscitation strategies could significantly reduce the risk of in-hospital mortality and decrease disease burden in patients with severe sepsis.^3–5^

Owing to a blunted erythropoietic response, diminished iron availability, and inhibitory effects of inflammatory cytokines, anemia is common among patients with severe sepsis.^6^ Inadequate oxygen delivery has also been reported in critically ill patients. According to the equation for calculating arterial blood oxygen delivery and oxygen content (oxygen delivery = 10 ∗ cardiac output ∗ (saturated arterial oxygen [SaO_2_] ∗ 1.39 ∗ hemoglobin [Hb] + 0.003 ∗ partial pressure of arterial oxygen [PaO_2_]), it seems reasonable that increasing the Hb level via transfusion would improve oxygen delivery.^7^ Rivers et al proposed a therapeutic protocol that included transfusion of packed red blood cells (pRBC) during the first 6 hours of resuscitation of anemic patients with septic shock in order to reduce in-hospital mortality.^3^ However, recent studies found that transfusion did not improve central venous oxygen saturation and oxygen utilization in anemic and septic patients.^8–10^

Practice guidelines for pRBC transfusion in anemic patients with severe sepsis remain controversial. The results of a multicenter, randomized controlled clinical trial of transfusion requirements in critical care did not support a liberal transfusion strategy for critically ill patients with euvolemic status who did not require active resuscitation.^11^ The latest international guidelines for the management of severe sepsis and septic shock also suggest pRBC transfusions only for patients with Hb concentrations below 7 g/dL in the absence of conditions such as myocardial ischemia, severe hypoxemia, acute hemorrhage, or ischemic coronary artery disease.^12^ Furthermore, a recent trial^13^ reported no mortality differences associated with higher transfusion Hb thresholds in patients diagnosed with septic shock.

However, these previous studies that did not observe positive effects of blood transfusion mostly evaluated effects in septic patients during the entire course of hospitalization rather during the early stages of resuscitation. In a multicenter study of 1036 patients with severe sepsis, Levy et al^14^ reported that early improvement in organ function (ie, in the first 24 hours) is closely associated with better health outcomes. Therefore, the effects of early blood transfusion during the first 24 hours of hospitalization in severe sepsis patients require further evaluation. In this retrospective cohort study, we analyzed a database from a tertiary university-affiliated hospital with a large patient volume in northern Taiwan to evaluate the association between mortality and early PRBC transfusion during the first 24 hours of hospitalization in patients with severe sepsis.

## MATERIALS AND METHODS

### Study Design

This retrospective cohort study assessed the effects of early blood transfusion on mortality and morbidity in ED patients with severe sepsis. The study protocol was approved by the institutional review board. Because of the retrospective nature of this study, the requirement for informed consent was waived.

### Study Setting and Population

**FIGURE 1:**
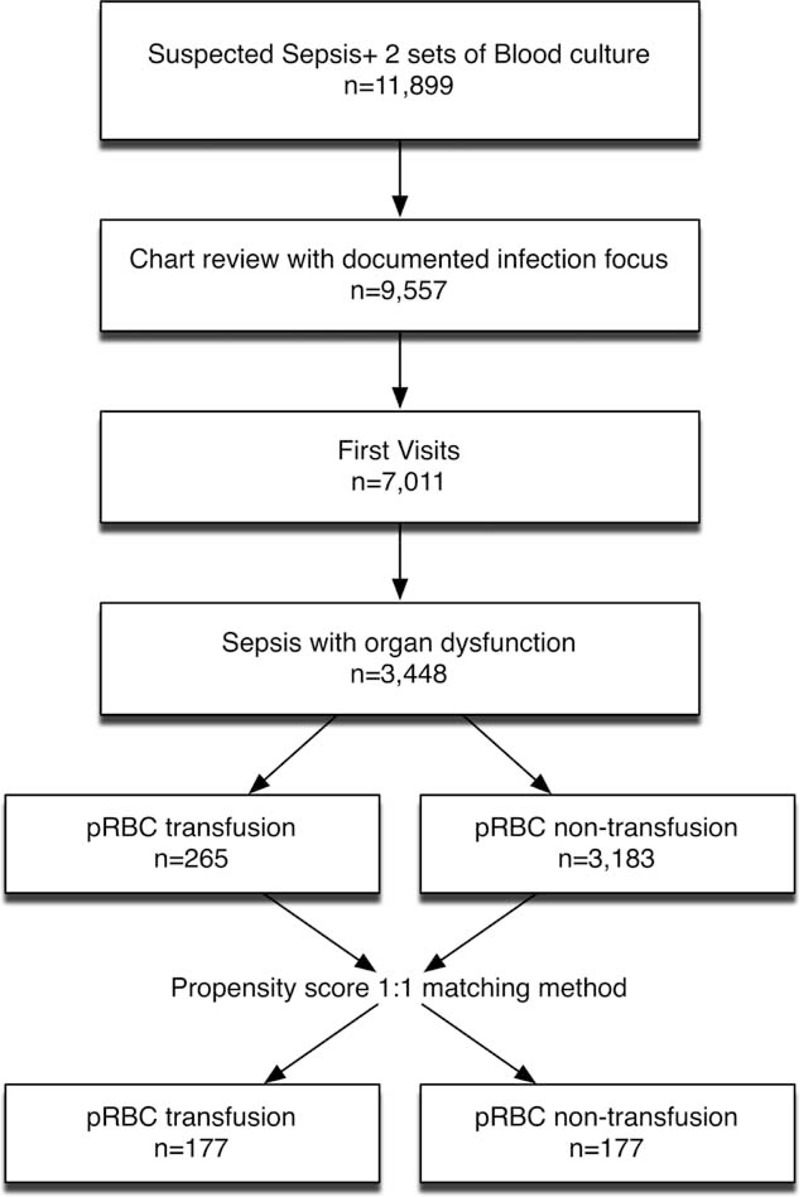
Study flow diagram.

This study was conducted in a tertiary medical center in northern Taiwan with 3700 beds and approximately 180,000 annual ED visits. This study adapted a 2-step inclusion strategy. In the 1st stage, all adult patients who visited the ED in 2010 with a documented diagnosis code of sepsis with at least 2 sets of blood cultures were selected for further chart review (Figure 1). After excluding patients transferred from other medical institutes, repeat visits, and patients with traumatic injuries, only the 1st ED visit was considered. In the 2nd stage, patients who fulfilled the criteria of severe sepsis were enrolled in order to evaluate the effectiveness of pRBC transfusion. The criteria of severe sepsis was modified from 2001 SCCM/ESICM/ACCP/ATS/SIS International Sepsis Definitions Conference, which was defined as the presence of septic shock or clinically diagnosed sepsis, plus evidence of organ dysfunction, including mean arterial pressure <70 mmHg, Glasgow coma scale < 13 points, estimated glomerular filtration rate <50 mL/min/1.73 m^2^ without previous history of chronic kidney disease, total bilirubin level ≥2 mg/dL, asparate aminotransferease (concentration ≥ 102 U/L, ammonia concentration ≥ 94 μmol/L, prothrombin time international normalized ratio ≥1.2, activated partial thromboplastin time ≥35.5 seconds, lactate level ≥2.2 mmol/L (or 19.8 mg/dL), platelet count <100 (10^3^/μL), or mechanical ventilation.^15^

### Data Collection

All medical records in the ED and during admission, including medical history, laboratory findings, radiologic images, and management notes, have been documented in an electronic database in our institution since 2004. Variables defined prior to data collection were entered in a standardized format during data collection. Trained research coordinators used predefined data collection forms to retrieve data from the electronic medical record using Structural Query Language (Microsoft Access, Redmond, WA). They also manually reviewed charts to confirm the results of the electronic chart review; all discrepant results were reviewed by a 3rd research coordinator and resolved by consensus.^16^ The data abstractors were blinded to the study objectives and hypotheses. Basic demographics, vital signs at ED triage, Glasgow coma scale scores, symptoms and signs, underlying illnesses, laboratory findings (including complete blood counts, differential counts, serum creatinine levels, liver function levels, bilirubin levels, serum sodium concentrations, serum potassium concentrations, C-reactive protein levels, procalcitonin levels, arterial blood gas analysis, lactate levels, and coagulation profiles), microbiological results, and discharge status were collected. The amount of pRBC transfused during the first 24 hours of hospitalization was recorded for each patient. In-hospital mortality was determined according to discharge status documented in the electronic medical record. The severity of sepsis was categorized according to Mortality in Emergency Department Sepsis (MEDS) scores.^17^

### Data Analysis

Chi-square and two-sample t- or Wilcoxon rank-sum tests were used to compare baseline characteristics between patients with and without pRBC transfusion. Propensity score (PS) matching was subsequently used to control for potential confounding after missing data was imputed and substituted with the single-imputed mean. The advantage of the propensity matching method is the 2-step analysis design, which enables a balance of possible confounding factors between the treated and control groups before “seeing” the results in the 1st step of the analysis. Furthermore, by using the PS generated from possible confounders, we examined the appropriateness of conventional multivariate regression methods by comparing different propensities of receiving pRBC transfusion in our treated and control groups, as described by Rubin.^18^ In brief, conventional regression-based methods were not recommended if the differences in standardized means, ratio of PS variances, and the ratio of the residuals of covariates over adjusting for PS between groups were smaller than 1 half or larger than 2. If the distribution of the covariates in both groups were symmetric, had nearly the same variance, or the sample sizes were approximately equal, conventional regression methods would be used to examine the causal relationship. The PS of a patient's probability of receiving transfusion was calculated according to multiple individual characteristics, including sex, age, underlying diseases, and laboratory results, vital signs via a modified step-wise logistic regression model in the 1st step of the analysis. In this model, we forced several clinically important confounders for the causal inference between transfusion and mortality to be kept in the model (Appendix), and used the Akaike information criterion to screen for additional potential predictors. In order to reduce heterogeneity among groups, different PS matching methods were considered, including exact, subclassification, nearest neighbor, optimal, and generic matching.^19,20^ Nearest neighbor matching without replacement with a ratio of 1:1 was chosen based on the percent balance improvement, defined as improvement of the mean difference between groups before and after matching. In the 2nd step of PS-matching-based analysis, the in-hospital mortality rates of the 2 groups were compared using multivariable logistic regression models to adjust for potential residual confounding “doubly robustly.”^21^ Multivariate logistic regression was used to adjust for possible residual confounding after matching as the some confounders were not balanced between groups. Based on cut-off thresholds recommended by international guidelines for management of severe sepsis and septic shock, a test for effect modification by different Hb levels of the association between transfusion and mortality was performed to compare the association of blood transfusion among patients with 3 different pretransfusion Hb levels: less than 7.0, between 7.0 and 9.0, and over 9.0 g/dL.^12^ All analyses were 2-tailed and *P*-values <0.05 were considered statistically significant. Analyses were performed using R (version 3.0.2; R Foundation for Statistical Computing, Vienna, Austria) and Stata (version 13.1; Stata Corp, College Station, TX).

## RESULTS

A total of 11,899 patients visited the ED during 2010 with a suspected clinical diagnosis of sepsis, as indicated by 2 sets of blood culture tests ordered by emergency physicians. After excluding 2546 transfers or repeat visits, 7011 patients had documented infection foci, among which 3448 met our definition for severe sepsis as clinical sepsis with evidence of organ dysfunction (Figure 1).

### Patient Characteristics

Among 3448 patients with severe sepsis or septic shock, 56% were male and more than half were older than 65 years of age (Table 1). The most common comorbidity was diabetes mellitus (n = 1843), followed by malignancy (n = 758) and cerebral vascular disease (n = 675). Among 371 patients who died during hospitalization, 206 were male, 238 were 65 years or older, and 180 were diabetic. Before adjusting the variables with PSs, 265 patients who received pRBC transfusions during the first 24 hours of hospitalization had a higher in-hospital mortality rate compared to the rate among those who did not receive transfusion (26% vs 9%, respectively, *P* < 0.001, Figure 1 and Table 1). Patients who received transfusions had lower mean arterial pressures (86 vs 98 mmHg) and Hb levels (7.6 vs 11.2 g/dL), and had more chronic kidney disease (12% vs 6%) and malignancy (32% vs 22%) than nontransfused patients. However, patients who received transfusion were more likely to have cardiovascular (25% vs 13%), renal (45% vs 43%), neurologic (34% vs 32%), hematologic (57% vs 35%), and metabolic organ dysfunction (12% vs 9%), despite similar sepsis severity as evaluated by MEDS scores (Table 1).

**TABLE 1:**
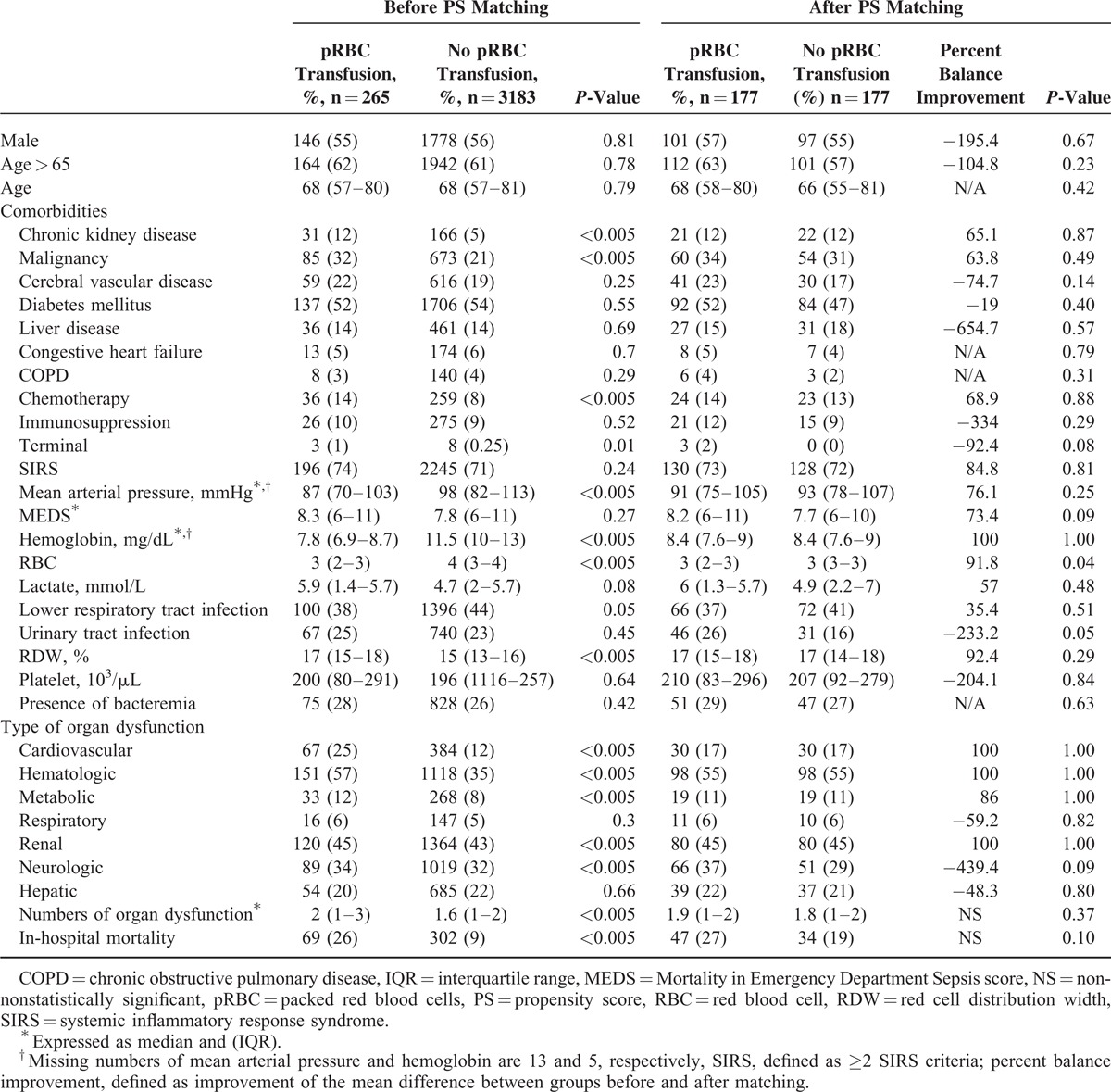
Characteristics of Patients Before/After Propensity Score Matching by Transfusion Status

### Propensity Score Matching

The differences in standardized means, ratio of variance of PS, and ratio of variances of residuals of covariates after adjusting for PSs between the transfused and nontransfused groups of patients were too different for conventional regression-based methods (1.41, 0.16, and 2.09, respectively). The asymmetric and different variances of the distribution of the covariates in both groups also supported this finding (Appendix). After comparing several different matching methods using PSs generated by nearly 30 variables retrieved from electronic charts, including one-to-many matching, tree-based, and subclassification-based methods, the 1:1 matching of 177 pairs of non-pRBC transfused to transfused patients minimized potential confounding between groups. After PS matching, the baseline characteristics of these 2 groups were nearly comparable (Table 1, Appendix Figure 1). After matching, there was no significant difference in in-hospital mortality between group of patients with and without pRBC transfusion (except for age and platelet level), although patients with pRBC transfusion were more likely to develop in-hospital mortality (27% vs 19%, odds ratio [OR] = 1.52, 95% CI: 0.92–2.51). We further adjusted for possible residual confounding between age and platelet level in order to assess the association between transfusion and in-hospital mortality in multivariate logistic regression analysis. Patients who received transfusions were associated with higher in-hospital mortality, although the difference was not statistically significant (OR = 1.52, 95% CI: 0.91–2.54). We did not find that the different Hb levels had any modification effect on the relationship between pRBC transfusion and in-hospital mortality (interaction *P* = 0.67).

## DISCUSSION

To our knowledge, this study is one of the largest to investigate the association between in-hospital mortality and early blood transfusion during the first 24 hours of hospitalization among patients with severe sepsis. This retrospective cohort study included 3448 patients with severe sepsis. Before matching, 265 patients received pRBC transfusion during the first 24 hours of their hospital stays; they tended to have a higher in-hospital mortality rate, lower Hb levels, lower mean arterial pressures, and higher likelihood of malignancy, chronic kidney disease and hematologic organ dysfunction while presenting at the ED. However, after adjusting for possible confounding factors and disease severity based on PS matching and multivariate logistic regression analysis, there was no significant difference in mortality among transfused and nontransfused patient groups.

The overall RBC transfusion rate during the first 24 hours of hospitalization in this retrospective cohort study was 7.6%, which was comparable to 2 recent Australasian Resuscitation In Sepsis Evaluation (ARISE) and Protocol-Based Care for Early Septic Shock (ProCESS) trials designed to test whether protocol-based care (early goal-directed therapy [EGDT]) was superior to usual care in septic shock patients.^22,23^ In the ProCESS study, 14.4% of patients in the protocol-based EGDT group received transfusion during the first 6 hours, compared to 8.3% in the protocol-based standard-therapy group, and 7.5% in the usual care group. However, the proportion of patients that received transfusions increased in the following 66 hours, with up to 20.9% of protocol-based standard-therapy patients and 18% of usual care patients receiving transfusions during the first 72 hours of hospitalization, compared to 19.8% in the protocol-based EGDT group. In the ARISE study, the transfusion rate was similar to that of the ProCESS study during the first 6 hours of hospitalization; 13.6% of patients in the EGDT group received transfusion during the first 6 hours, compared to 7% in the usual care group. The medical institution in which our study was conducted currently has no universal protocol for the treatment of severe sepsis. In other words, the ED physicians in our study provided nonprotocol-based usual care to severe sepsis patients, which could explain why the transfusion rate in our study was similar to rates in the usual care groups in the ARISE and ProCESS studies.

The currently available evidence from clinical trials has conflicting results regarding the efficacy of early blood transfusion for severely septic patients. The EGDT proposed by Rivers et al recommends pRBC transfusion for anemic patients to increase the Hb levels up to 10 g/dL who do not respond to aggressive fluid and inotropic agent resuscitation during the first 6 hours of resuscitation. However, the transfusion requirements in critical care and septic shock (Transfusion requirements in septic shock trial) trials did not observe significant differences in mortality rates for pRBC transfusions performed for higher versus lower thresholds of Hb levels within 72 hours after ICU admission or during their ICU stay, respectively.^11,13,24^ Nevertheless, none of the following clinical trials focused on the effect of early blood transfusion; they examined only the overall transfusion rate for patients with critical illnesses. Therefore, it remains unclear whether early transfusion of pRBC or bundle care for severe sepsis contributes to reduced mortality.

Observational studies, sometimes more generalizable than clinical trials, have also reported conflicting results of different transfusion practices for patients with critical illnesses. A growing number of studies have attempted to determine the effectiveness of transfusion for patients with severe sepsis.^8,25–27^ Two large prospective cohort studies performed in Europe (the Anemia and Blood Transfusion in Critical Care) and the US (the Anemia and Blood transfusion in the Critically Ill) reported an association between blood transfusion and poor clinical outcomes.^25,28^ However, only 38% of patients in the PS matched subgroup in the Anemia and Blood Transfusion in Critical Care cohort and 11% in the Anemia and Blood transfusion in the Critically Ill study were septic. On the other hand, the Sepsis Occurrence in Acutely Ill Patients study analyzed PS-matched patients and found a better 30-day survival rate among patients transfused with leukodepleted pRBC.^29^ Nonetheless, in the Sepsis Occurrence in Acutely Ill Patients study, more than half of the participants were surgical patients; only one-fourth had severe sepsis or septic shock on admission to ICU. It is reasonable that surgical patients with acute blood loss may benefit more from transfusion. In another similar study, Park et al^8^ analyzed propensity-matched septic patients and found blood transfusion to be associated with lower mortality rates. These researchers used the registration system to select their study population, which might introduce selection bias due to voluntary participation. In contrast, our study utilized electronic medical records for data retrieval to consecutively include all ED patients clinically diagnosed with severe sepsis.

There are several potential explanations for the differences between our study findings and those of previous observational studies. First, we focused on early pRBC transfusion for severe sepsis patients in the first 24 hours of hospitalization rather than during their entire hospital stay. In a small subgroup of patients who were transfused within the first 24 hours in a retrospective cohort study by Fuller et al^30^ (n = 14), there were also no significant differences in in-hospital mortality rates between groups. Second, leukodepleted pRBC transfusion for critically ill patients is not practiced in our institution, which could be another reason for the differences in results. Last, our patients had an overall lower in-hospital mortality rate compared to other study populations. The different spectrum of patients with severe sepsis could also explain why blood transfusion might have different effects on their outcomes.

## LIMITATIONS

Our study has several limitations. First, although PS matching can adjust for most known and recorded confounding factors, the risk for potential unmeasurable confounding factors inevitably exists in observational studies. For example, patients that appear to be clinically ill may not be recorded in the medical record and are more likely to receive pRBC at the treating physician's discretion. Second, compared to other studies, our study population had lower in-hospital mortality and moderate sepsis severity as indicated by MEDS scores. Accordingly, we might need to reconsider recommendations for pRBC transfusion in all patients with “severe” sepsis, as the results of our study do not support the practice for patients with less severe illness. Third, indication bias is a common limitation of observational studies; while many sophisticated statistical methods including the PS matching have been developed to adjust for measurable confounders, remains an inherited bias that could potentially influence the testing of causal relationships. In order to minimize possible confounding results due to indication bias, our study simulated scenarios that clinicians might face in making decisions to transfuse patients based on laboratory values, vital signs, and underlying illnesses. Fourth, since there is no reference test for sepsis, we chose a pragmatic definition based on physician discretion and 2 sets of blood cultures obtained in the ED, similar to other studies.^17^ However, our final analysis included only patients with severe sepsis or septic shock in order to evaluate the effectiveness of pRBC transfusion. Therefore, we expect the influence of the imperfect definition of sepsis to be minimal. Fifth, only vital signs obtained during the ED triage were used to define severe sepsis or septic shock. It is possible that the patients developed worsening vital signs during the first 24 hours of their ED visits, which were not included in our final analysis. Therefore, we caution the readers to generalize our results to patients whose stage of sepsis might be early. Last, the low proportion of patients receiving transfusions in our study (3%) was much lower than the previously reported 40% among sepsis patients in intensive care units (ICUs), which could result in selection bias in our study. However, in our PS matching process, some patients who received transfusions were not matched to nontransfused patients because of the extreme likelihood for them to be transfused. This is one of the benefits of PS matching rather than traditional regression methods.

## CONCLUSIONS

In this study, patients with severe sepsis who received nonleukodepleted pRBC transfusion had higher mortality and increased likelihood of chronic kidney disease and hematologic organ dysfunction, along with lower Hb levels and mean arterial pressures before adjusting for possible confounding factors. However, after PS matching and multivariate regression to adjust for potential confounding factors, the mortality rate did not differ significantly between patients who had and had not received transfusions during the first 24 hours of their hospitalization. In other words, early pRBC transfusion may not improve survival in patients with severe sepsis. Randomized controlled studies are necessary to confirm the association between early blood transfusion and mortality in severe sepsis patients.

## Supplementary Material

Supplemental Digital Content
