# Supplementary material for: STROBE-compliant article: Blood Transfusions within the First 24 Hours of Hospitalization Did Not Impact Mortality Among Patients with Severe Sepsis
Source: Medicine (Baltimore). 2016 Jan 29;95(4):e2601. doi: 10.1097/MD.0000000000002601 (PMC5291581; doi:10.1097/MD.0000000000002601)

**Appendix**

**Recursive partitioning algorithm** (variables selection for rpart in R )

m.out.fix.rpart<- matchit(prbc_update~sirs+meds+organ_cv+organ_neu+organ_ren+organ_hem+organ_hep+organ_met+lti+uc+organ_res+chemo+ckd+immunosuppression+malignancy+terminal+male+age+map+rdw+plt+hb+hct+rbc,data=dta.all,ratio=1,method= "nearest",exact=c("hb","organ_cv","organ_hem"))

**Figure A1. jitter plot**


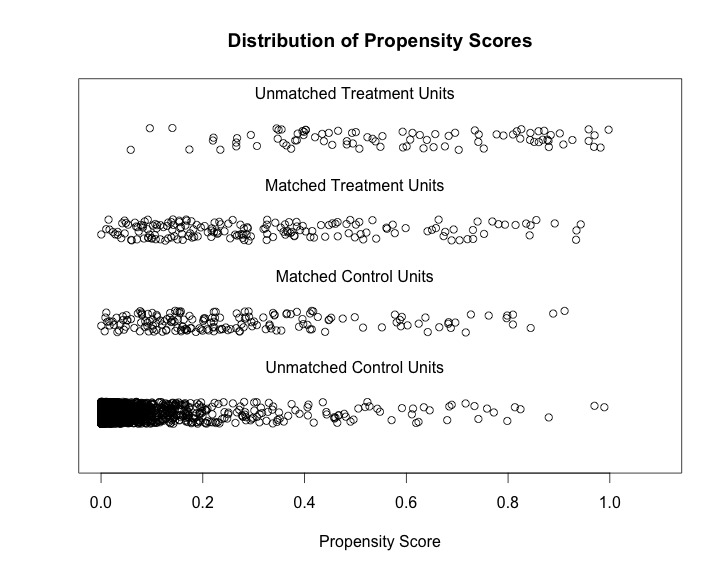


**Figure A2. histogram**


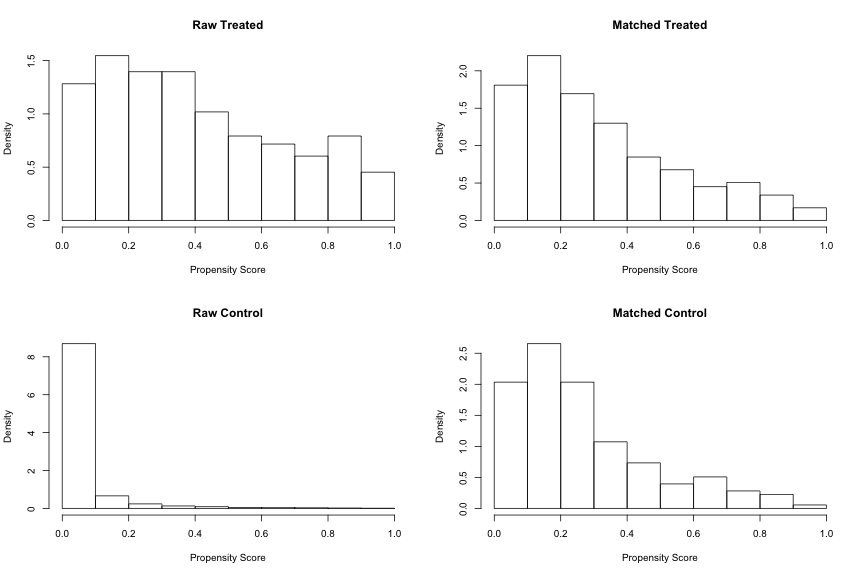


**Figure A3 qq plot**


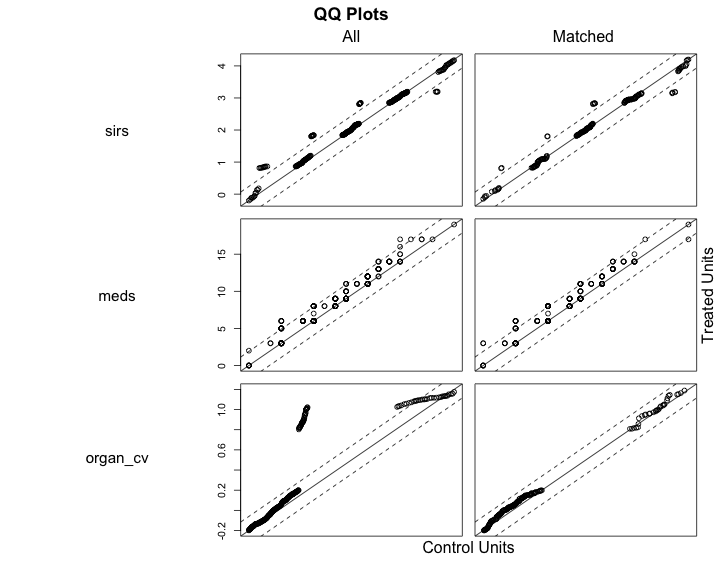


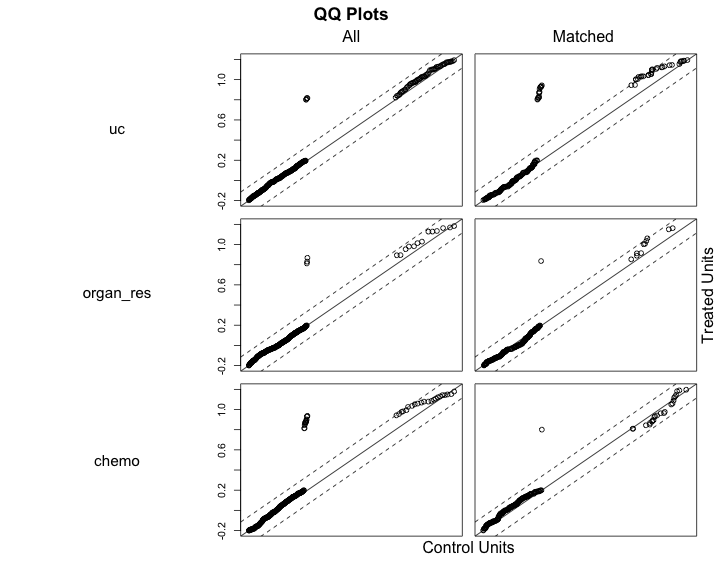


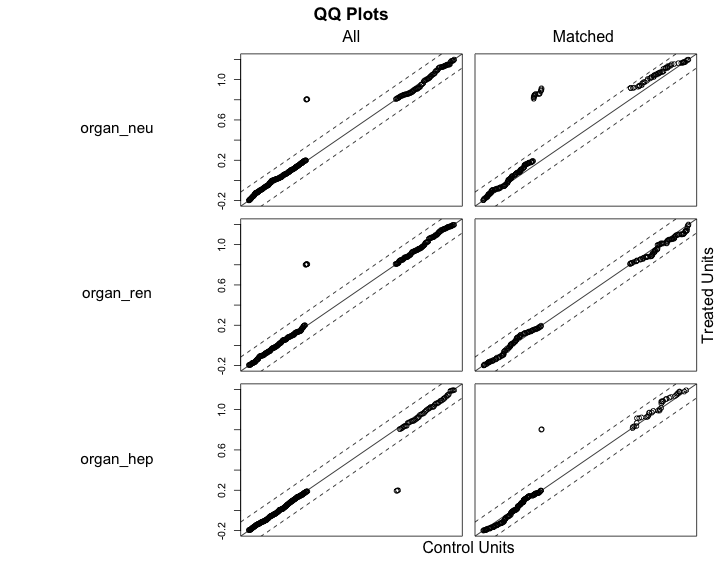

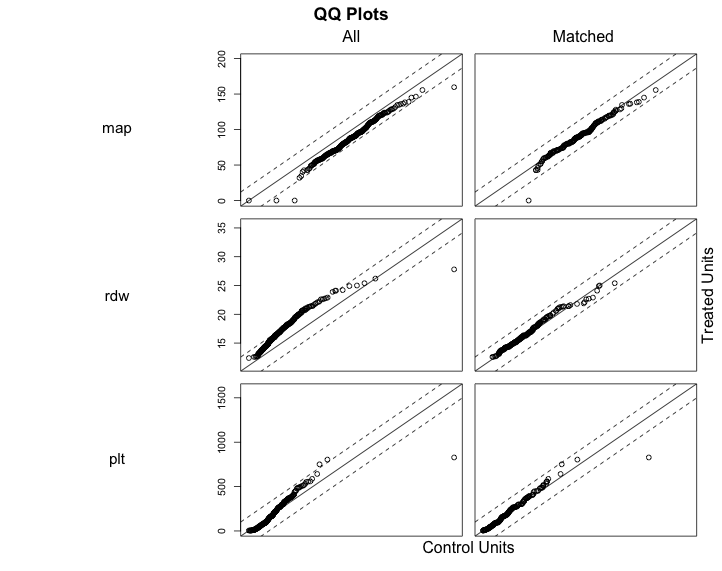

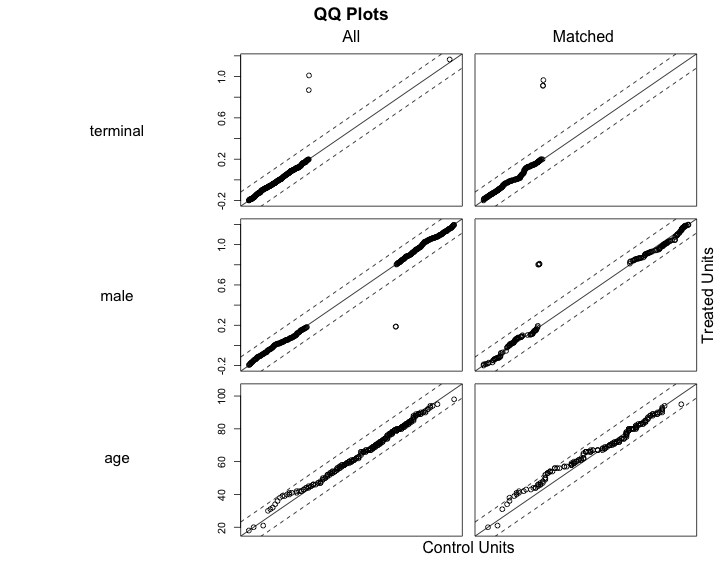


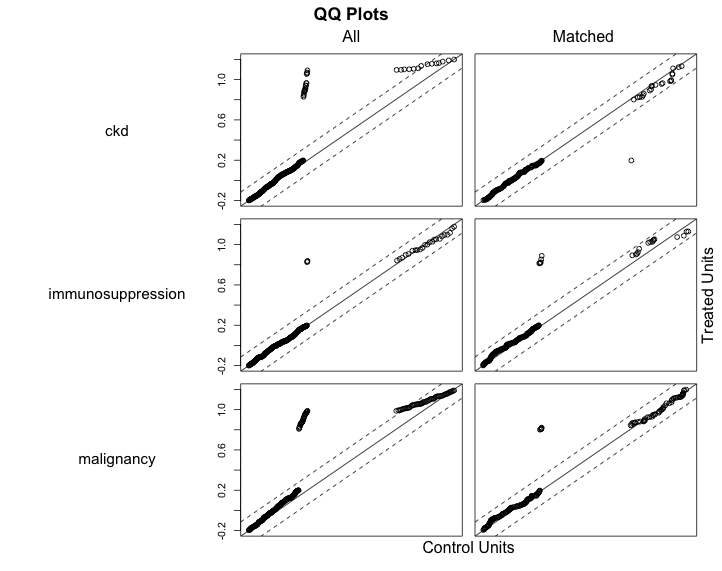

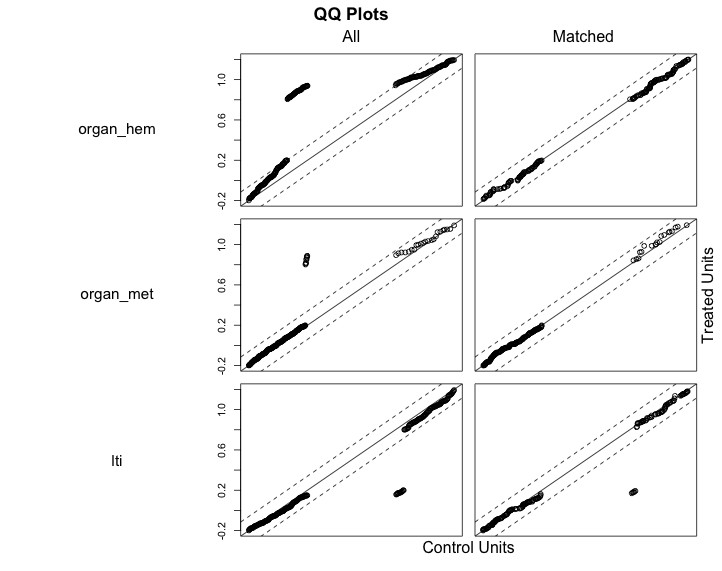


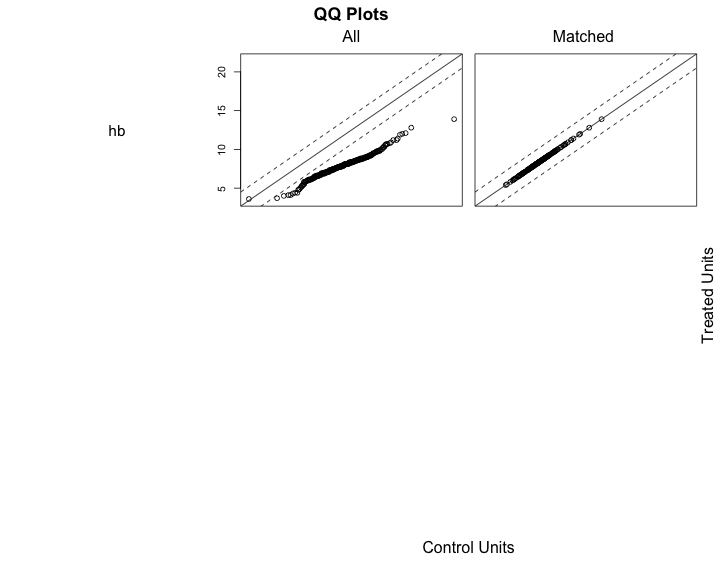

Supplement: Supplemental Digital Content [file medi-95-e2601-s001.docx]
